# Supplementary material for: Unveiling the influence of social network characteristics on sustainable performance in small and medium-sized enterprises: A dynamic capabilities perspective
Source: PLoS One. 2025 Oct 16;20(10):e0325378. doi: 10.1371/journal.pone.0325378 (PMC12530530; doi:10.1371/journal.pone.0325378)
Supplement: Attachment — Submitted filename: Responce to Reviewers.docx [file pone.0325378.s002.docx]

**Editor：**

1. Language and Readability: Both reviewers emphasized the need for thorough proofreading. The current manuscript contains numerous grammatical issues, long and convoluted sentences, and awkward phrasing. This significantly detracts from the clarity and professionalism of the paper. I recommend working with a professional language editor to revise the manuscript for readability and coherence.

We sincerely appreciate the Editor's valuable suggestions. In response to the feedback, we have engaged a professional language editor to enhance the manuscript's readability and coherence.

2. Abstract and Introduction: The abstract should be revised to clearly state the study's purpose, motivation, and key findings. Similarly, the introduction lacks a clear explanation of the research motivation and the paper's contribution to the field. Please include a well-defined research question and discuss your contribution to the literature, as suggested by Reviewer 2.

Informed by expert advice, social networks are recognized as pivotal in modern business operations. They enable enterprises to build meaningful interactions with customers, partners, and employees, gain insights into market demands and feedback, identify new collaborators, and jointly explore markets or drive innovation. Resource bricolage refers to the process by which enterprises leverage their internal strengths and resources, integrate external opportunities, and create a tailored resource system to meet their specific needs. This approach is particularly critical for resource-constrained enterprises, as it empowers them to respond flexibly to market fluctuations and environmental challenges, enhancing adaptability and innovation. Given these dynamics, exploring the interplay between social network characteristics, resource bricolage, and the sustainable performance of SMEs holds significant theoretical and practical value. This exploration forms the primary motivation for this research. The revised summary, aligned with expert recommendations, is as follows:

*Gaining a deeper understanding of the mechanism through which social networks influence sustainable performance and exploring the role of resource bricolage in enhancing competitiveness can help small and medium-sized enterprises (SMEs) better adapt to market changes in the digital age and achieve sustainable development. In the context of accelerated technological change and resource scarcity, the question of how SMEs can leverage network resources to achieve sustainable growth has become a pressing issue. This study, grounded in social network theory and resource-based theory, develops a theoretical model exploring the relationships between social network characteristics, resource bricolage, and SME sustainable performance. Using survey data from 230 SMEs, the study empirically demonstrates that both social network scale and social network strength have significant positive impacts on sustainable performance. Additionally, resource bricolage mediates the effects of social network scale and strength on SME performance. Moreover, opportunity identification and opportunity exploitation—key dimensions of dynamic capabilities—moderate the relationship between resource bricolage and sustainable performance. This study highlights the mechanisms through which SMEs can leverage social networks to enhance their sustainable performance, offering theoretical guidance for effectively identifying and utilizing network resources in uncertain and dynamic environments.*

*Contribution：*

*The contributions of this study are as follows: Based on resource-based theory and social network theory, this research explores the relationship between social network structure, resource bricolage, and sustainable performance. It also tests the boundary effects of dynamic capabilities on the relationship between resource bricolage and sustainable performance, thereby expanding the theoretical applicability in different contexts. This study strongly aligns with the perspective proposed by scholar Wang ( ), which emphasizes the importance of network resources in sustaining a firm's competitive advantage.。*

3. Theoretical Framework: The theoretical model (Figure 1) needs further clarification. Reviewer 1 highlighted that the edges and their direction in the figure are unclear. A detailed explanation of the relationships between constructs should be added to the text. Moreover, before diving into the hypotheses, provide an overview of the theoretical analysis section for better comprehension.

We sincerely thank the experts for their valuable suggestions on improving the content of this article. In response to the recommendations provided by Reviewer 1, we have revised the theoretical framework diagram by refining the variable boundaries and using arrows to clearly indicate the directions of relationships. Additionally, we have incorporated explanations of the relationships between constructs into the main text. The detailed explanation is as follows:

*On the left side of the diagram are the independent variables, representing the characteristics of social networks, which include two dimensions: social network scale and social network strength. The middle section depicts the mediating variable, representing the degree of resource bricolage. On the right side are the dependent variables, representing organizational sustainable performance. At the top of the diagram are the moderating variables, representing dynamic capabilities, which consist of two dimensions: opportunity identification capability and opportunity exploitation capability.*

Finally, before examining the hypothesis, we provide an overview of the theoretical analysis section, which is as follows:

*Our study, based on social network theory and resource-based theory, develops a theoretical model linking social network characteristics with sustainable performance. It also tests the mediating role of resource bricolage and the moderating effect of dynamic capabilities. The proposed theoretical hypotheses are as follows*

4. Sampling and Methodology: The description of the sampling technique and the details of the questionnaire are insufficient. Reviewer 1 pointed out missing information about the sampling procedure and the questionnaire’s contents. Please provide detailed information about how the sample was selected, the representativeness of the sample, and the contents of the questionnaire. Tables detailing the sample characteristics and data collection process should be included for clarity.

We are very sorry for the insufficient description of Sampling techniques and questionnaire details in the Sampling and Methodology section. Based on this, we added the description of sampling techniques and questionnaire details, including sample selection process, sample representativeness and questionnaire content. Description is as follows :

*Before the survey, the research group explained the contents and objetctives of the survey to the companies. After obtaining their consent , the research group distributed questionnaires to the senior executives and staff from the performance departments of the participating companies. The questionnaire consists of two parts. The first part focus on demographic information, including gender, age, educational background, enterprise size and years of establishment, and its influence on enterprise performance. The second part examed variables such as the characteristics of enterprise social network, resource bricolage, sustainable performance and dynamic capability (See Table 1 for details on variables). A total of 230 companies participated in the survey, Out of 300 questionnaires distributred, 220 were retrieved, and the response rate reached 73.3%.*

*5.*Data Presentation and Analysis: Several tables, especially Table 3, are unclear and need to be restructured. Additionally, statistical results should be better explained. Reviewer 1 mentioned the need to explain model fit indices and the reliability coefficients in more detail. For instance, what does Cronbach's alpha of 0.916 imply for the study's reliability?

To ensure clarity and precision, we have revised all the charts and conducted a thorough analysis of the statistical results, including model fit indices and reliability and validity coefficients as suggested by Reviewer 1. All the charts have been presented in the final manuscript, and the statistical results are explained below.

*Considering the contextual specificity of enterprise, the study modifies the measurement of key variables based on well-established scale from interntional literature. Pre-testing is conducted to refine the descriptions and language of the scale items, ensuring reliability and validity, thereby enhancing the applicability of the research findings. The questionnaire employs a seven-point Likert scale for responses, with specific details as follows.*

*Social Network Characteristics: Based on the views of Watson [29] and Tan [30], as well as the work of Luo and Park et al. [31], two representative dimensions, social network scale and social network strength are selected for measurement. The scale of social network consists of five items, with a Cronbach's α of 0.916. The social network strength was measured using six items, with a Cronbach's α of 0.911. Experts generally consider internal reliability insufficient if Cronbach's α is below 0.6, acceptable when it falls between 0.7 and 0.8, and strong when it ranges from 0.8 to 0.9. These resuts indicates a high reliability for themeasurement of social network characteristcs.*

*Resource Bricolage: by referring to the research of Davidsson et al. [32], the resource bricolage scale includs six items that assess the integration of existing resources to address with challenges and leverage opportunities to solve new problems. The scale achieves a Cronbach's α of 0.915，indicating high reliability.*

*Entrepreneurial Dyamic Capability: Based on the research of Ma et al. [33] and Foss et al. [34], focusing on opportunity recognition capability encompass the ability to quickly gather entrepreneurial opportunity information and identify the impact of new information,, with a scale reliability of Cronbach's α= 0.891. Opportunity exploitation ability includes six aspects, such as the ability to develop new products and services, the ability to develop new market areas, with a Cronbach's α of 0.895. Both the opportunity recognition and exploitation capability demonstrate good reliability.*

*Sustainable Performance: Based on the research of Zhu and Fei [35], to measure sustainable performance,,, which mainly includes enterprise growth performance and profitability performance. Items include metrics such as employees growth and new products and services growth. The Cronbach's α of this scale is 0.928, indicating that it has good reliability.*

5. Literature Positioning and Contribution: As Reviewer 2 suggested, the paper's contribution to the literature needs to be articulated more clearly. This can be achieved by citing recent relevant studies (e.g., DOI: 10.1016/j.physa.2019.123174) and positioning your findings within the broader context of social network theory and resource-based theory. Clearly outline what gap in the literature this paper fills.

Thanks very much for the experts' opinions. In view of the background research of social network theory and resource-based theory, with reference to the latest relevant research, we quoted the latest relevant literature, strengthened the introduction of theoretical contributions in the introduction, and filled in some research gaps. The theoretical contributions in the introduction are as follows:

*The contributions of this study are as follows: Based on resource-based theory and social network theory, this research explores the relationship between social network structure, resource bricolage, and sustainable performance. It also tests the boundary effects of dynamic capabilities on the relationship between resource bricolage and sustainable performance, thereby expanding the theoretical applicability in different contexts. This study strongly aligns with the perspective proposed by scholar Wang ( ), which emphasizes the importance of network resources in sustaining a firm's competitive advantage.*

**Reviewer 1:**

1. The sentences are too long which makes it very hard to comprehend. For example, in Abstract: “Based on social network theory and resource-based theory, a theoretical model of social network characteristics, resource patchwork and small and medium enterprises (SMEs) sustainable performance represented by social network scale and social network strength was constructed, and 220 questionnaire data were collected.” It is advised to break down such sentences into two or even three sentences for readability.

We sincerely thank the experts for their valuable suggestions. In response, the authors have thoroughly revised the abstract and introduction sections, adjusting and optimizing sentence structures to enhance readability.The theoretical contributions in the introduction are as follows:

Abstract: *Gaining a deeper understanding of the mechanism through which social networks influence sustainable performance and exploring the role of resource bricolage in enhancing competitiveness can help small and medium-sized enterprises (SMEs) better adapt to market changes in the digital age and achieve sustainable development. In the context of accelerated technological change and resource scarcity, the question of how SMEs can leverage network resources to achieve sustainable growth has become a pressing issue. This study, grounded in social network theory and resource-based theory, develops a theoretical model exploring the relationships between social network characteristics, resource bricolage, and SME sustainable performance. Using survey data from 230 SMEs, the study empirically demonstrates that both social network scale and social network strength have significant positive impacts on sustainable performance. Additionally, resource bricolage mediates the effects of social network scale and strength on SME performance. Moreover, opportunity identification and opportunity exploitation—key dimensions of dynamic capabilities—moderate the relationship between resource bricolage and sustainable performance. This study highlights the mechanisms through which SMEs can leverage social networks to enhance their sustainable performance, offering theoretical guidance for effectively identifying and utilizing network resources in uncertain and dynamic environments.*

Introduction: *Social networks play a crucial role in the modern business operations of enterprises. Through social networks, businesses can establish deep interactions with customers, partners, and employees, gain insights into market demands and feedback, identify new partners, and collaborate on market expansion or innovation. The diversified information exchange platforms provided by social networks facilitate the acquisition and sharing of knowledge and information, thereby driving innovation. However, social networks also present certain challenges and risks, such as the rapid spread of information, the ease with which negative information can proliferate, and the difficulty in managing online reputations.The healthy development of enterprises as an effective lever for driving the transformation and upgrading of economic structure. To achieve this goal, pursuing high-tech and high value-added industries is undoubtedly essential. However, issues such as deviations in enterprise integration concepts, insufficient innovation drive, low levels of integration among key plays, limited military-civilian techology transfer, and inadquate innovation resources significantly hider the long-term development of enterprises [1], These challenges undermine the industrial foundation necessary to support enterprise growth. Consequently, enterprises experience phenomena such as "bubblelization" and "homogenization", with a severe decline in the interactive efects among stakeholders. In order to mitigate the negative impact of resources scarcity on the enterprise growth, it is crucial for enterprises to explore rational pathway for resource acquisition.*

*In recent years, academic research on enterprise development performance have deepened, covering topics such as enterprise concepts, development status, and evolutionary processes [2,3], business development models and performance evaluation [4,5], corporate financing and resource allocation [6,7] and the efficiency of techology transfer and its influencing factors [8-10]. However, empirical research on the mechanism and pathways through which enterprise performance operates remain insufficient. Given the significant role that enterprise development plays in promoting national defense economic growth, maintaining social stability, and expanding employment, it is essential to explore the factors influencing enterprise performance within the context of China.*

*Under the influence of technological barriers, enterprises face obstacles in the process of technological development due to limited innovation resources. Innovation resources in enterprises ten to evolve rapidly, with some resources depreciating quickly and being non-renewable, lending to inefficiency and resource waste [11]. The key to overcoming these challenges lies in collaborating with other innovation partners and the acquistion of resources for innovative development [12]. In the field of entrepreneurship, existing studies employs diverse methodologies, including case studies, meta-analysis, and empirical analysis based on primary or secondary data [13] to examine the impact of social network on enterprise growth [14], organizational behavior [15], and performance [16]. These studies also explore the effect of factors such as emotional trust [17], environmental uncertainty [18] and other complex entrepreneurial scenarios on capabilities like knowledge integration, resource acquisition, risk perception, all of which influence enterprise development.*

*In summary, extensive social networks enable enterprises to access more resources, with businesses positioning themselves as the central node within these networks to achieve mutual benefits through resource sharing, information exchange, and technology integration. However, some scholars have raised concerns, suggesting that the expansion in the scale of social network may, to some content, affect the network’s stability, potentially having adverse effects on the enterprises performance and development. The close ties between enterprises and other social entities make networks more tightly interconnected, thus reducing cooperation risks. Yet, the question remains whether such close communication could burden enterprises, which could negatively impact performance and development. In the context of new challenges, the influence of strong and weak network relationship on enterprise performance warrants further investigation. Based on these issues, this study asserts the necessity of examining whether and how the characteristics of social network affect enterprise performance.*

*Resource bricolage is characterized by innovation, flexibility, and the ability to take immediate action. It helps reduce innovation risks and costs while continuously generating new ideas and business opportunities for enterprises. Through resource bricolage, enterprises can break and restructure existing business channels and cooperation networks, creating a more flexible and diverse business structure. This process not only lowers enterprise risk but also enhances adaptability in an ever-changing environment. Most scholars have explored the impact of resource bricolage on enterprise resources, considering it as a mediating variable that influences innovation and development at the micro level. However, researches on its role in the innovation development of mature enterprises remain relatively limited. Enterprises often face resources scarcity, and bricolage behavior facilitates cooperation with other social entities, The impact of this on enterprise performance requires further investigation.*

*To summary, based on social network theory and resource-based theory, this study develops a "Characteristics-Behavior-Performance" theoretical model. It primarily explores how enterprises, facing resources constraints, can leverage connections with other social entities within their networks to acquire the heterogeneous resources necessary for innovation, thereby uncovering the core mechanism of network structure. In terms of research methodology, case studies and grounded theory typically focus on investigating a single enterprise or a small number of enterprises, and the generalizability of the finding may be limited. Therefore, this study will select representative enterprises from different regions of China --- such as the eastern, central, and western regions --- and conduct a detailed investigation of their innovation performance at the micro level. This approach enriches the development of entrepreneurship theory.*

*From the perspective of current research, the research on social network and enterprise performance is relatively abundant [19]. However, due to the plight of small and medium-sized enterprises (SMEs) facing threats to their survival due to insufficient resources [20], there is still a gap in the research on social network and sustainable performance of SMEs with SMEs as the main body of research. In addition, from the perspective of resource patchwork, the existing literature has not been answered to explore the mechanism of social network and sustainable performance.The contributions of this study are as follows: Based on resource-based theory and social network theory, this research explores the relationship between social network structure, resource bricolage, and sustainable performance. It also tests the boundary effects of dynamic capabilities on the relationship between resource bricolage and sustainable performance, thereby expanding the theoretical applicability in different contexts. This study strongly aligns with the perspective proposed by scholar Wang [21], which emphasizes the importance of network resources in sustaining a firm's competitive advantage.*

*The second section of the study provides a theoretical analysis and proposes research hypotheses. The third section outlines the research design, including sample selection, data collection, variable definition, and data testing. The fourth section discusses the research findings and offers managerial implications; the fifth section addresses the limitations of the study and suggests directions for future research.*

5. The abstract is a little unclear. The purpose of the study should be defined in a much clearer way.

Thanks to expert advice, social networks play a crucial role in modern business operations. Through social networks, enterprises can establish in-depth interactions with customers, partners and employees, understand market needs and feedback, find new partners, and jointly explore markets or innovate. Resource patchwork refers to the enterprise through the use of their own advantages and resources, the use of external opportunities and resources, combined into a resource system suitable for their own needs, in order to enhance the competitiveness of enterprises. For enterprises with limited resources, resource patchwork is particularly important. It enables enterprises to flexibly respond to the challenges of market changes and business environment, and improve the adaptability and innovation ability of enterprises. Based on this, it is of theoretical and practical significance to explore the mechanism between social network characteristics, resource patchwork and sustainable performance of smes, which is also the research motivation of this paper. Revised in accordance with expert opinion, the summary is as follows:

*Gaining a deeper understanding of the mechanism through which social networks influence sustainable performance and exploring the role of resource bricolage in enhancing competitiveness can help small and medium-sized enterprises (SMEs) better adapt to market changes in the digital age and achieve sustainable development. In the context of accelerated technological change and resource scarcity, the question of how SMEs can leverage network resources to achieve sustainable growth has become a pressing issue. This study, grounded in social network theory and resource-based theory, develops a theoretical model exploring the relationships between social network characteristics, resource bricolage, and SME sustainable performance. Using survey data from 230 SMEs, the study empirically demonstrates that both social network scale and social network strength have significant positive impacts on sustainable performance. Additionally, resource bricolage mediates the effects of social network scale and strength on SME performance. Moreover, opportunity identification and opportunity exploitation—key dimensions of dynamic capabilities—moderate the relationship between resource bricolage and sustainable performance. This study highlights the mechanisms through which SMEs can leverage social networks to enhance their sustainable performance, offering theoretical guidance for effectively identifying and utilizing network resources in uncertain and dynamic environments.*

6. The same suggestion as above for the Introduction.

According to the opinions of experts, we have sorted out the introduction part again, and added the research framework arrangement and theoretical contribution at the end of the introduction. The revised introduction content is as follows:

*Social networks play a crucial role in the modern business operations of enterprises. Through social networks, businesses can establish deep interactions with customers, partners, and employees, gain insights into market demands and feedback, identify new partners, and collaborate on market expansion or innovation. The diversified information exchange platforms provided by social networks facilitate the acquisition and sharing of knowledge and information, thereby driving innovation. However, social networks also present certain challenges and risks, such as the rapid spread of information, the ease with which negative information can proliferate, and the difficulty in managing online reputations.The healthy development of enterprises as an effective lever for driving the transformation and upgrading of economic structure. To achieve this goal, pursuing high-tech and high value-added industries is undoubtedly essential. However, issues such as deviations in enterprise integration concepts, insufficient innovation drive, low levels of integration among key plays, limited military-civilian techology transfer, and inadquate innovation resources significantly hider the long-term development of enterprises [1], These challenges undermine the industrial foundation necessary to support enterprise growth. Consequently, enterprises experience phenomena such as "bubblelization" and "homogenization", with a severe decline in the interactive efects among stakeholders. In order to mitigate the negative impact of resources scarcity on the enterprise growth, it is crucial for enterprises to explore rational pathway for resource acquisition.*

*In recent years, academic research on enterprise development performance have deepened, covering topics such as enterprise concepts, development status, and evolutionary processes [2,3], business development models and performance evaluation [4,5], corporate financing and resource allocation [6,7] and the efficiency of techology transfer and its influencing factors [8-10]. However, empirical research on the mechanism and pathways through which enterprise performance operates remain insufficient. Given the significant role that enterprise development plays in promoting national defense economic growth, maintaining social stability, and expanding employment, it is essential to explore the factors influencing enterprise performance within the context of China.*

*Under the influence of technological barriers, enterprises face obstacles in the process of technological development due to limited innovation resources. Innovation resources in enterprises ten to evolve rapidly, with some resources depreciating quickly and being non-renewable, lending to inefficiency and resource waste [11]. The key to overcoming these challenges lies in collaborating with other innovation partners and the acquistion of resources for innovative development [12]. In the field of entrepreneurship, existing studies employs diverse methodologies, including case studies, meta-analysis, and empirical analysis based on primary or secondary data [13] to examine the impact of social network on enterprise growth [14], organizational behavior [15], and performance [16]. These studies also explore the effect of factors such as emotional trust [17], environmental uncertainty [18] and other complex entrepreneurial scenarios on capabilities like knowledge integration, resource acquisition, risk perception, all of which influence enterprise development.*

*In summary, extensive social networks enable enterprises to access more resources, with businesses positioning themselves as the central node within these networks to achieve mutual benefits through resource sharing, information exchange, and technology integration. However, some scholars have raised concerns, suggesting that the expansion in the scale of social network may, to some content, affect the network’s stability, potentially having adverse effects on the enterprises performance and development. The close ties between enterprises and other social entities make networks more tightly interconnected, thus reducing cooperation risks. Yet, the question remains whether such close communication could burden enterprises, which could negatively impact performance and development. In the context of new challenges, the influence of strong and weak network relationship on enterprise performance warrants further investigation. Based on these issues, this study asserts the necessity of examining whether and how the characteristics of social network affect enterprise performance.*

*Resource bricolage is characterized by innovation, flexibility, and the ability to take immediate action. It helps reduce innovation risks and costs while continuously generating new ideas and business opportunities for enterprises. Through resource bricolage, enterprises can break and restructure existing business channels and cooperation networks, creating a more flexible and diverse business structure. This process not only lowers enterprise risk but also enhances adaptability in an ever-changing environment. Most scholars have explored the impact of resource bricolage on enterprise resources, considering it as a mediating variable that influences innovation and development at the micro level. However, researches on its role in the innovation development of mature enterprises remain relatively limited. Enterprises often face resources scarcity, and bricolage behavior facilitates cooperation with other social entities, The impact of this on enterprise performance requires further investigation.*

*To summary, based on social network theory and resource-based theory, this study develops a "Characteristics-Behavior-Performance" theoretical model. It primarily explores how enterprises, facing resources constraints, can leverage connections with other social entities within their networks to acquire the heterogeneous resources necessary for innovation, thereby uncovering the core mechanism of network structure. In terms of research methodology, case studies and grounded theory typically focus on investigating a single enterprise or a small number of enterprises, and the generalizability of the finding may be limited. Therefore, this study will select representative enterprises from different regions of China --- such as the eastern, central, and western regions --- and conduct a detailed investigation of their innovation performance at the micro level. This approach enriches the development of entrepreneurship theory.*

*From the perspective of current research, the research on social network and enterprise performance is relatively abundant [19]. However, due to the plight of small and medium-sized enterprises (SMEs) facing threats to their survival due to insufficient resources [20], there is still a gap in the research on social network and sustainable performance of SMEs with SMEs as the main body of research. In addition, from the perspective of resource patchwork, the existing literature has not been answered to explore the mechanism of social network and sustainable performance.The contributions of this study are as follows: Based on resource-based theory and social network theory, this research explores the relationship between social network structure, resource bricolage, and sustainable performance. It also tests the boundary effects of dynamic capabilities on the relationship between resource bricolage and sustainable performance, thereby expanding the theoretical applicability in different contexts. This study strongly aligns with the perspective proposed by scholar Wang [21], which emphasizes the importance of network resources in sustaining a firm's competitive advantage.*

*The second section of the study provides a theoretical analysis and proposes research hypotheses. The third section outlines the research design, including sample selection, data collection, variable definition, and data testing. The fourth section discusses the research findings and offers managerial implications; the fifth section addresses the limitations of the study and suggests directions for future research.*

7. Section: “Theoretical analysis and research hypothesis”. Before going into the subsection, an overview of the section must be provided.

Thanks very much for the input of the experts, we provide an overview of this part before investigating the hypothesis, which is as follows:

*Our study, based on social network theory and resource-based theory, develops a theoretical model linking social network characteristics with sustainable performance. It also tests the mediating role of resource bricolage and the moderating effect of dynamic capabilities. The proposed theoretical hypotheses are as follows*

8. “Based on this, this paper will explore the scale and strength of social networks from two aspects.” What are the two aspects?

Thank you for this comment. We are very sorry for your confusion due to our poor choice of words. This refers to the theoretical discussion of social network characteristics from two dimensions of social network scale and social network strength. We delect this sentence and replace it in another sentence.

9. The theoretical model presented in Fig 1 is unclear. What do the edges indicate? Is there any direction associated with the edges? Fig 1 must be explained in detail.

According to the opinions of experts, the author re-modified the contents of Figure 1, including the determination of the boundary and arrow direction, as shown in the Figure 1.

*Among them, the left side of the picture is the independent variable, which represents the characteristics of social network, including two dimensions of social network size and social network strength. The middle of the picture is the intermediate variable, representing the resource patchwork. The right side of the picture is the dependent variable, which represents the sustainable performance of the enterprise. The upper part of the picture is divided into adjustment variables, representing dynamic capability, including two dimensions of opportunity identification capability and opportunity development capability.*

10. What does the following sentence mean: “In line with the key and typical characteristics of case selection.”?

I am very sorry for the trouble caused to the expert reading. It is intended to show that the selection of sample enterprises is representative. The changes are as follows:

*The selected sample reflects a high level of representatives.*

11. In Sample Selection, every representative enterprise must be mentioned.

Based on the opinions of experts, we have listed the representative companies in different fields as follows:

*Our study selected a sample of 230 representative small and medium-sized enterprises (SMEs) from various industries, including food and biopharmaceuticals (e.g., Guyu Biotechnology Co., Ltd.), metallurgical machinery (e.g., Hengan Intelligent Control Technology Co., Ltd.), materials (e.g., New Material Technology Co., Ltd.), and new energy (e.g.f Fengfa Technology Development Co., Ltd.). Since their establishment, these companies has experienced challenges such as resource shortage and environmental volatility. Through establishing strong networks with other external stakeholders, they have achieved significant scientifical technological innovation outcomes. The selected sample reflectes a high level of representativeness.*

12. “First of all, understand the basic situation of the enterprise and the problems of enterprise performance.” What does it mean？

We delect the unclear sentence.

13. “During the investigation, senior leaders and staff of performance departments were asked to adjust and modify the contents of the questionnaire.” What questionnaire? What are the contents of the questionnaire? What parameters were considered when designing the questionnaire?

We sincerely appreciate the expert's valuable suggestions. The description in this section was not sufficiently clear, and we have revised and reorganized it. The updated content is as follows:

*Before the survey, the research group explained the contents and objetctives of the survey to the companies. After obtaining their consent , the research group distributed questionnaires to the senior executives and staff from the performance departments of the participating companies. The questionnaire consists of two parts. The first part focus on demographic information, including gender, age, educational background, enterprise size and years of establishment, and its influence on enterprise performance. The second part examed variables such as the characteristics of enterprise social network, resource bricolage, sustainable performance and dynamic capability (See Table 1 for details on variables). A total of 230 companies participated in the survey, Out of 300 questionnaires distributred, 220 were retrieved, and the response rate reached 73.3%.*

14. “The research team went deep into the sample enterprises to investigate.” How many sample enterprises?

In this study, there are 220 sample enterprises.

15. Section “Data collection and sample situation”, paragraph 2: The statistics should be presented in the form of a table.

Thank you very much for the expert's advice. This part was deleted before due to the word limit. Now the author has added the explanation again.

| Table 1 Demographic description of samples. | | | |
| --- | --- | --- | --- |
| Variable | Feature | Number | Proportion(%) |
| Gender | Male | 125 | 56.8 |
|  | Female | 95 | 43.2 |
| Age | <=20 | 0 | 0 |
|  | 21-30 | 98 | 44.5 |
|  | 31-40 | 102 | 46.4 |
|  | 41-50 | 10 | 4.5 |
|  | 51-60 | 8 | 3.6 |
|  | Over 60 years old | 2 | 1 |
| Education | Primary school | 4 | 1.8 |
|  | Junior high school | 15 | 6.9 |
|  | Senior high school | 26 | 11.8 |
|  | Undergraduate course | 167 | 75.9 |
|  | Postgraduate degree | 8 | 3.6 |
| Enterprise scale | <=50 | 55 | 25 |
|  | 51-200 | 105 | 47.7 |
|  | 201-500 | 49 | 22.3 |
|  | 501 and above | 11 | 5 |
| Age of establishment of enterprise | Less than 5 years | 104 | 47.3 |
|  | 5 to 8 years | 61 | 27.7 |
|  | 8-10 years | 48 | 21.8 |
|  | More than 10 years | 7 | 3.2 |

16. Section “Empirical Analysis”. Same suggestion as given in comment 9.

Thank you very much for the expert's opinion, the revised content is as follows:

*In this study, confirmatory factor analysis (CFA) was conducted using AMOS22.0 to test the structure validity of the questionnarie Initially, SPSS22.0 was used to calculate the Kaiser-Meyer-Olkin (KMO) value and Bartlett’s test of sphericity for the questionnaire population. The results showed that the overall KMO value was 0.929, exceeding the standard threshold of 0.7. Then, single-factor, two-factor, three-factor, four-factor, five-factor and six-factor models were constructed using the principal component analysis method. The fit indices of each model were analyzed to verify the structural validity of the questionnaire. The results showed that compared with other models, the six-factor model proposed in this study exhibited the best fit (CMIN/DF=1.791, RMSEA（Root Mean Square Error of Approximation）=0.06, SRMR（Standardized Root Mean Square Residual） = 0.054,CFI（comparative fit index）=0.925, GFI（goodness-of-fit index）=0.817, TLI（Tucker-Lewis index）=0.917) . All fit indices met the standards.*

17. “Considering the situational particularity of the enterprise, the measurement of key variables is modified on the basis of the mature scale abroad, and the description and language of the scale items are modified through the pretest, so as to ensure the reliability and validity of the scale and make the research conclusions more applicable.” The sentence is way too long. Extremely hard to follow and comprehend.

Thanks very much for the expert's advice, we will sort out this part of the content again to enhance the readability of the article. The changes are as follows:

*Considering the contextual specificity of enterprise, the study modifies the measurement of key variables based on well-established scale from interntional literature. Pre-testing is conducted to refine the descriptions and language of the scale items, ensuring reliability and validity, thereby enhancing the applicability of the research findings.*

18. “The scale of social network was measured with 5 item scales, and the reliability coefficient was 0.916.” What are the 5 item scales? Where are they listed? What is the definition of the reliability coefficient? What does the number 0.916 mean here? What is the scale of the reliability coefficient? What are the good, bad and neutral values of the reliability coefficient?

Thanks very much for the opinions of experts, the revised content is as follows:

*Social Network Characteristics: Based on the views of Watson [32] and Tan [33], as well as the work of Luo and Park et al. [34], two representative dimensions, social network scale and social network strength are selected for measurement. The scale of social network consists of five items, with a Cronbach's α of 0.916. The social network strength was measured using six items, with a Cronbach's α of 0.911. Experts generally consider internal reliability insufficient if Cronbach's α is below 0.6, acceptable when it falls between 0.7 and 0.8, and strong when it ranges from 0.8 to 0.9. These resuts indicates a high reliability for the measurement of social network characteristics.*

19. “The social network strength was measured by a 6-item scale with a reliability coefficient of 0.911.” Same questions as above.

Thanks very much for the opinions of experts, the revised content is as follows:

*The scale of social network consists of five items, with a Cronbach's α of 0.916. The social network strength was measured using six items, with a Cronbach's α of 0.911. Experts generally consider internal reliability insufficient if Cronbach's α is below 0.6, acceptable when it falls between 0.7 and 0.8, and strong when it ranges from 0.8 to 0.9. These resuts indicates a high reliability for the measurement of social network characteristics.*

20. The same questions for resource bricolage, entrepreneurial dynamic capability and enterprise performance.

Thanks very much for the opinions of experts, the revised content is as follows:

*Resource Bricolage: by referring to the research of Davidsson et al. [35], the resource bricolage scale includes six items that assess the integration of existing resources to address with challenges and leverage opportunities to solve new problems. The scale achieves a Cronbach's α of 0.915，indicating high reliability.*

*Entrepreneurial Dyamic Capability: Based on the research of Ma et al. [36] and Foss et al. [37], focusing on opportunity recognition capability encompass the ability to quickly gather entrepreneurial opportunity information and identify the impact of new information, with a scale reliability of Cronbach's α Of 0.891. Opportunity exploitation ability includes six aspects, such as the ability to develop new products and services, the ability to develop new market areas, with a Cronbach's α of 0.895. Both the opportunity recognition and exploitation capability demonstrate good reliability.*

*Sustainable Performance: Based on the research of Zhu and Fei [38], to measure sustainable performance, which mainly includes enterprise growth performance and profitability performance. Items include metrics such as employees growth and new products and services growth. The Cronbach's α of this scale is 0.928, indicating that it has good reliability.*

21. Is reliability coefficient the “Cronbach's a coefficient” as indicated afterwards in Section “Reliability and validity analysis”? I think the authors refer to Cronbach's alpha coefficient. The authors are advised to give complete details of this coefficient.

Yes, the reliability coefficient is the "Cronbach's a coefficient." We made a supplementary explanation on Cronbach's alpha coefficient, which is as follows:

*Cronbach's a coefficient is an important index to measure reliability. In general, the higher the Klonbach coefficient, the higher the reliability. In basic research, the reliability should be at least 0.80 to be considered acceptable; In exploratory studies, a confidence level of 0.70 is acceptable. Confidence between 0.70 and 0.98 is considered high confidence, while confidence below 0.35 is considered low confidence and should be considered rejected.*

22. “the mean variance variation AVE”. Do the authors mean variance extracted?

That means “Average Variance Extracted”，at the same time, the author has made corrections in the article.

23. The authors are advised to provide full form of an acronym where it is used first (for example, KMO, SRMR, etc.).

According to the opinions of experts, we provide the full spelling of the abbreviation, and the revised content is as follows:

*KMO（Kaiser-Meyer-Olkin）= 0.929, RMSEA（Root Mean Square Error of Approximation）=0.06, SRMR（Standardized Root Mean Square Residual） = 0.054,CFI（comparative fit index）=0.925, GFI（goodness-of-fit index）=0.817, TLI（Tucker-Lewis index）=0.917)*

24. The n-factor models should first be discussed in detail and then the results should be presented. In its current form, it is extremely confusing.

Thank you very much for the advice of the experts. According to the advice of the experts, we have optimized the content of the table to make the content clear. Where X1 refers to the scale of social networks, X2 refers to the strength of social networks, M refers to the resource patchwork of intermediary variables, T1 refers to the ability to identify opportunities, T2 refers to the ability to develop opportunities, and Y refers to sustainable performance. These variables correspond to each other in the previous article, so the table uses letters instead.

| Table 2 Results of confirmatory factor analysis | | | | | | | | | |
| --- | --- | --- | --- | --- | --- | --- | --- | --- | --- |
| model | χ² | df | χ²/ df | RMSEA | CFI | GFI | TLI | IFI | SRMR |
| Six-factor model | 859.551 | 480 | 1.791 | 0.06 | 0.925 | 0.817 | 0.917 | 0.925 | 0.054 |
| Five-factor model^1^ | 1462.791 | 485 | 3.016 | 0.096 | 0.806 | 0.635 | 0.789 | 0.807 | 0.084 |
| Five-factor model^2^ | 1242.637 | 485 | 2.562 | 0.084 | 0.85 | 0.757 | 0.836 | 0.851 | 0.087 |
| Four-factor model | 1845.173 | 489 | 3.773 | 0.113 | 0.731 | 0.598 | 0.709 | 0.733 | 0.102 |
| Three-factor model | 2671.639 | 492 | 5.43 | 0.142 | 0.567 | 0.495 | 0.535 | 0.57 | 0.199 |
| Two-factor model | 1518.304 | 208 | 5.921 | 0.15 | 0.517 | 0.468 | 0.484 | 0.52 | 0.143 |
| Single factor model | 1856.902 | 209 | 6.815 | 0.163 | 0.428 | 0.423 | 0.39 | 0.432 | 0.152 |
| Note：Six-factor model: (X1,，X2，M，T1，T2, Y);  Five-factor model ^1:^ (X1+X2，M，T1，T2, Y)；  Five-factor model ^2^: (X1，X2，M，T1+T2);  Four-factor model:（X1+X2，M，T1+T2, Y);  Three-factor model:（X1+X2，M+T1+T2, Y）;  Two-factor model: (X1+X2+M+T1+T2, Y);  Single factor model:(X1+X2+M+T1+T2+Y) | | | | | | | | | |
|  |  |  |  |  |  |  |  |  |  |
|  |  |  |  |  |  |  |  |  |  |
|  |  |  |  |  |  |  |  |  |  |
|  |  |  |  |  |  |  |  |  |  |
|  |  |  |  |  |  |  |  |  |  |

25. “The results showed that compared with other models, the six-factor model constructed in this study (CMIN/DF=1.791, RMSEA=0.06, SRMR = 0.054,CFI=0.925, GFI=0.817, TLI=0.917) had the best fitting indexes, and all fitting indexes met the standard.” So, what does this result entail?

According to the opinions of experts, we added the description of the model fit index in the paper, which is as follows:

*In general, a CMIN/DF value closer to 1 indicates a better model fits. Smaller RMSEA and SRMR values signify improved fit, while higher CFI, GFI, TLI values reflect better model performace. These results indicate that the six-factor model constructed in this study demonstrates the most favorable fit, supporting the validity of the theoretical model.*

26. Table 3 is a mess and cannot be understood.

In questionnaire analysis, the analysis of common method bias is an important step to ensure the accuracy and reliability of research results. Table 3 shows the results of the common method bias test for testing. After discussion by the authors, we only discuss the text, and no longer present this table.

27. While I understand that p is the p-value, what is r?

Here r is the coefficient of correlation between variables.

30. Tables need to be placed round about where they are discussed. Besides, authors are advised not to spread the table over two pages. This needs to be managed.

Thanks to the expert's advice, we have optimized the layout of the table and adjusted the content of the table so that it is no longer than 1 page.

**Reviewer 2:**

Reviewer #2: This article addresses the issue of social network characteristics and resource particles that affect the sustainable performance of small and medium enterprises within the framework of social network theory and resource-based theory. The study needs to be revised according to the following points:

- 1. There are many errors in the English of the study, it should be reviewed. 2. The motivation of the study should be explained in the abstract section.

Thanks to expert advice, social networks play a crucial role in modern business operations. Through social networks, enterprises can establish in-depth interactions with customers, partners and employees, understand market needs and feedback, find new partners, and jointly explore markets or innovate. Resource patchwork refers to the enterprise through the use of their own advantages and resources, the use of external opportunities and resources, combined into a resource system suitable for their own needs, in order to enhance the competitiveness of enterprises. For enterprises with limited resources, resource patchwork is particularly important. It enables enterprises to flexibly respond to the challenges of market changes and business environment, and improve the adaptability and innovation ability of enterprises. Based on this, it is of theoretical and practical significance to explore the mechanism between social network characteristics, resource patchwork and sustainable performance of smes, which is also the research motivation of this paper. Revised in accordance with expert opinion, the summary is as follows:

*Gaining a deeper understanding of the mechanism through which social networks influence sustainable performance and exploring the role of resource bricolage in enhancing competitiveness can help small and medium-sized enterprises (SMEs) better adapt to market changes in the digital age and achieve sustainable development. In the context of accelerated technological change and resource scarcity, the question of how SMEs can leverage network resources to achieve sustainable growth has become a pressing issue. This study, grounded in social network theory and resource-based theory, develops a theoretical model exploring the relationships between social network characteristics, resource bricolage, and SME sustainable performance. Using survey data from 230 SMEs, the study empirically demonstrates that both social network scale and social network strength have significant positive impacts on sustainable performance. Additionally, resource bricolage mediates the effects of social network scale and strength on SME performance. Moreover, opportunity identification and opportunity exploitation—key dimensions of dynamic capabilities—moderate the relationship between resource bricolage and sustainable performance. This study highlights the mechanisms through which SMEs can leverage social networks to enhance their sustainable performance, offering theoretical guidance for effectively identifying and utilizing network resources in uncertain and dynamic environments.*

- 3.There should be a space before the references in the text. For example [11], [12], [13], [14], [16], etc.

Thanks to the advice of the experts, we have readjusted the format of the references in the paper.

- 4. Some studies that have attracted attention in recent years on social networks should be cited in the introduction section. Eg, doi: 10.1016/j.physa.2019.123174, 10.1126/science.abp9364, 10.1016/j.ijinfomgt.2023.102621

Thanks very much for the opinions of experts, we have carefully read the relevant literature of social network and sustainable performance research, and quoted the literature proposed by experts and other cutting-edge literature to enrich the theoretical research.

- 5. The organization of the paper should be added as a paragraph at the end of the introduction section.

Thank you very much for your constructive comments. In the revised manuscript, we have added a paragraph about organization of the paper into the Introduction section (see pages 8). Specifically, we wrote:

*The second section of the study provides a theoretical analysis and proposes research hypotheses. The third section outlines the research design, including sample selection, data collection, variable definition, and data testing. The fourth section discusses the research findings and offers managerial implications; the fifth section addresses the limitations of the study and suggests directions for future research.*

- 6. The contribution of this research to the literature should be clearly discussed in the introduction.

Thanks very much for the experts' opinions. In view of the background research of social network theory and resource-based theory, with reference to the latest relevant research, we quoted the latest relevant literature, strengthened the introduction of theoretical contributions in the introduction, and filled in some research gaps. The theoretical contributions in the introduction are as follows:

*From the perspective of current research, the research on social network and enterprise performance is relatively abundant [19]. However, due to the plight of small and medium-sized enterprises (SMEs) facing threats to their survival due to insufficient resources [20], there is still a gap in the research on social network and sustainable performance of SMEs with SMEs as the main body of research. In addition, from the perspective of resource patchwork, the existing literature has not been answered to explore the mechanism of social network and sustainable performance.The contributions of this study are as follows: Based on resource-based theory and social network theory, this research explores the relationship between social network structure, resource bricolage, and sustainable performance. It also tests the boundary effects of dynamic capabilities on the relationship between resource bricolage and sustainable performance, thereby expanding the theoretical applicability in different contexts. This study strongly aligns with the perspective proposed by scholar Wang [21], which emphasizes the importance of network resources in sustaining a firm's competitive advantage.*
