# Supplementary material for: Unveiling the influence of social network characteristics on sustainable performance in small and medium-sized enterprises: A dynamic capabilities perspective
Source: PLoS One. 2025 Oct 16;20(10):e0325378. doi: 10.1371/journal.pone.0325378 (PMC12530530; doi:10.1371/journal.pone.0325378)
Supplement: Attachment — Submitted filename: Response to Reviewers.docx [file pone.0325378.s003.docx]

**Reviewer 1:**

Reviewer #1: Overall, the authors have largely revised the paper and according to my suggestions and comments. There still are some persisting and new issues (minor) which the authors should address. My comments to address these issues are given below:

1. In Abstract, the first sentence is again too long to comprehend. Again, it is advised to break the sentence down. The authors are also advised to do this throughout the paper wherever applicable.

**According to the expert's opinion, we changed the sentence as follows:**

*Gaining a deeper understanding of the mechanism through which social networks influence sustainable performance and exploring the role of resource bricolage in enhancing competitiveness are valuable. These insights can help small and medium-sized enterprises (SMEs) better adapt to market changes in the digital age and ultimately, this can enable SMEs to achieve sustainable development.*

**Similarly, we modified other sentences with similar problems in the article:**

**Original text:** *Under the influence of technological barriers, enterprises face obstacles in the process of technological development due to limited innovation resources. Innovation resources in enterprises ten to evolve rapidly, with some resources depreciating quickly and being non-renewable, lending to inefficiency and resource waste [11].*

**Revised sentence:** *Technological barriers often hinder enterprises' technological development due to limited innovation resources. These resources evolve rapidly, with some depreciating quickly or becoming non-renewable. As a result, enterprises face inefficiencies and resource waste, which further constrain their innovation capabilities.*

**Original text:** *In addition, entrepreneurial dynamic capabilities enhance and organization’s sensitivity to fluctuations in the external environment, enabling the integration of strategic planning capabilities to address environmental changes and promptly adjust production and operational strategies [30].*

**Revised sentence:** *Furthermore, entrepreneurial dynamic capabilities strengthen an organization’s ability to detect and respond to external environmental fluctuations. By integrating strategic planning capabilities, organizations can effectively adapt to environmental changes and quickly adjust their production and operational strategies.*

**Original text:** *Taking social network scale and strength as representative characteristics of social network structure, the empirical research finds that an expaned social network scale - characterized by broader business scope and diverse service targets - stimulate the demand for resource richness and promotes resource bricolage behaviors.*

**Revised sentence:***The empirical research uses social network scale and strength as key indicators of social network structure. It finds that an expanded social network scale—reflected by a broader business scope and diverse service targets—increases the demand for resource richness. This, in turn, encourages resource bricolage behaviors.*

2. In Introduction, last paragraph, when the authors write, “The second section…”, the authors should instead write something like this: “The rest of the paper is organized as follows. Section “Theoretical Background and Hypotheses Development” provides a theoretical analysis and proposes research hypotheses.”

**Thanks to the valuable advice of the experts, we have rearranged the last paragraph of the introduction, which is as follows:**

*The rest of the paper is organized as follows. Section “Theoretical Background and Hypotheses Development” provides a theoretical analysis and proposes research hypotheses. Section "Methodology" introduces the source of sample data, the measurement process of variables, etc., and analyzes the data results in detail. Section “Discussion and Conclusion” discusses the research findings and offers managerial implications.*

3. The authors must add the contributions of the study in bullet points in Introduction. Possibly before the last paragraph in Introduction.

**Thanks very much for the experts' opinions. In view of the background research of social network theory and resource-based theory, with reference to the latest relevant research, we quoted the latest relevant literature, strengthened the introduction of theoretical contributions in the introduction, and filled in some research gaps. The theoretical contributions in the introduction are as follows:**

*From the perspective of current research, the research on social network and enterprise performance is relatively abundant ^[19]^. However, due to the plight of small and medium-sized enterprises (SMEs) facing threats to their survival due to insufficient resources ^[20]^, there is still a gap in the research on social network and sustainable performance of SMEs with SMEs as the main body of research. In addition, from the perspective of resource patchwork, the existing literature has not been answered to explore the mechanism of social network and sustainable performance.The contributions of this study are as follows: Based on resource-based theory and social network theory, this research explores the relationship between social network structure, resource bricolage, and sustainable performance. It also tests the boundary effects of dynamic capabilities on the relationship between resource bricolage and sustainable performance, thereby expanding the theoretical applicability in different contexts. This study strongly aligns with the perspective proposed by scholar Wang ^[21]^, which emphasizes the importance of network resources in sustaining a firm's competitive advantage.*

4. In “Theoretical Background and Hypotheses Development”, the last sentence of paragraph one, must be changed to something like, “In the subsequent sections, we discuss the proposed theoretical hypotheses.”

**According to the expert's opinion, we changed the sentence as follows:***“In the subsequent sections, we discuss the proposed theoretical hypotheses”.*

5. “Based on this, this study will explore the size and strength of social networks from two aspects.” Again, what are the two aspects? The authors in their response seem to suggest the two aspects are size and strength of social networks. So, the sentence must be changed accordingly. Presently, the sentence creates an unnecessary confusion.

**Thank you very much for the expert opinion, the two aspects are size and strength of social networks. According to the expert's opinion, we changed the sentence as follows:**

*“Based on this, this study will explore the size and strength of social networks”.*
